# Low-Temperature Ionic Layer Adsorption and Reaction Grown Anatase TiO_2_ Nanocrystalline Films for Efficient Perovskite Solar Cell and Gas Sensor Applications

**DOI:** 10.1038/s41598-018-29363-0

**Published:** 2018-07-20

**Authors:** Shoyebmohamad F. Shaikh, Balaji G. Ghule, Umesh T. Nakate, Pritamkumar V. Shinde, Satish U. Ekar, Colm O’Dwyer, Kwang Ho Kim, Rajaram S. Mane

**Affiliations:** 1 School of Physical Sciences, Swami Ramanand Teerth Marathwada University, Nanded, 431 606 India; 2 School of Chemistry, University College of Cork, Cork, T12 YN 60 Ireland; 3 Micro-Nano Systems Centre, Tyndall National Institute, Lee Maltings, Cork, T12 R5CP Ireland; 4 Environmental Research Institute, University College Cork, Lee Road, Cork, T23 XE10 Ireland; 5 Hybrid Material Solution National Core Research Center, Pusan National University, Busan, 600-735 Republic of Korea

**Keywords:** Organic-inorganic nanostructures, Metal-organic frameworks

## Abstract

A low-temperature (90 °C) and directly grown anatase titanium dioxide (TiO_2_) nanocrystalline film using successive ionic layer adsorption and reaction (SILAR) for perovskite solar cell and gas sensor applications. TiO_2_ nanocrystalline electron transfer layer (ETL) improves the power conversion efficiency (PCE) of perovskite solar cells due to faster charge transport kinetics as well as slower charge recombination process. The optimized TiO_2_ nanocrystalline ETL (15 L) demonstrates as high as ~10% PCE with a short circuit current density of 18.0 mA/cm^2^, open circuit voltage of 0.81 V and fill factor of 66.3% in perovskite solar cells. Furthermore, room-temperature ammonia sensing characteristics of TiO_2_ nanocrystalline film (25 L) were  demonstrated for various concentration levels of ammonia in dry air conditions. A high room-temperature response of 80% was achieved at 100 ppm of ammonia with rapid response and recovery signatures of 30 and 85 s, and nearly fifteen days stability, respectively. The response of the sensor to other gases such as formaldehyde, petrol, ethanol acetone, and ammonia etc, indicated a high selectivity towards volatile organic compounds of ammonia gas. The room temperature operation, with high selectivity, repeatability and fast transition times, suggests potentially useful in flexible and cost-effective production in optoelectrochemical device technology.

## Introduction

In the past few decades, transition metal oxides have attracted tremendous attention in scientific and technological research due to their unique electrical, optical, and mechanical properties^1–3^. Among transition metal oxides, TiO_2_ plays a vital role because of its chemical stability, and inexpensive and non-toxic signatures. TiO_2_ maintains efficient charge transport capability even with a relatively wide band-gap (~3–3.35 eV), and high specific surface area in many of its nanoscale morphologies^4–6^. The anatase phase of TiO_2_ is largely preferred in photovoltaics, solar cell and gas sensing applications, because of its superior electron mobility and chemical stability compared with rutile and brookite phases^7^. Gopal *et al*., reported the Ti-O phase diagram and concluded that anatase TiO_2_ is more stable than the rutile phase at room-temperature (27 °C) and atmospheric pressure (760 mmHg)^8^. During the past several decades, control in the formation of TiO_2_ film with specific morphology for the purpose of improving solar cells and low-temperature gas sensor performance has proven the benefits of TiO_2_. Extremely great number of methods developed in literature for the preparation of TiO_2_ nanocrystalline films containing nanowires^9^, nanotubes^10^, nanorods^11^, and hollow microspheres^12^, etc., morphologies included spray pyrolysis^13^, sol-gel^14^, wet chemical^15^, electrodeposition^16^, magnetron sputtering^17^, and chemical vapor deposition^18^, etc. These methods are being undertaken to direct synthesize TiO_2_ nanocrystalline films on conducting/non-conducting substrates for both perovskite solar cell and gas sensor applications. The vast majority of reported methods involve either long deposition times or complicated processing steps, with lack of clarity in the roles of some of the synthetic steps in these growth processes. The valuable works motivated us to handle this challenge in the synthetic route and applications potential, such as the use of successive ionic layer adsorption and reaction (SILAR) technique. In spite of its simplicity, SILAR has a number of advantages: unlike physical technique, SILAR does not require high quality target and/or substrate nor it does require vacuum at any stage; the deposition rate and the thickness of the film can be easily controlled over a wide range by changing the deposition cycles; there are virtually no restrictions on substrate material, dimensions or its surface profile; moreover, it is convenient for large area direct deposition, which helps to enhance interconnection and adherence of film with superior properties^19^.

In this work, we unveil SILAR-mediated binder-free chemical synthesis of phase pure anatase TiO_2_ films directly grown on flourine-tin-oxide (FTO) and soda-lime glass (SLG) substrates for perovskite solar cell and chemical gas sensor applications. By tuning the SILAR cycles, anatase TiO_2_ nanocrystalline films grown at low-temperature (90 °C) on FTO were compatible with a sequentially deposited and full coverage perovskite layer^20^. As a result, the perovskite solar cells based on this TiO_2_ ETL demonstrate a PCE of ~10%, which is the one of the best figures for directly grown TiO_2_ ETL-based perovskite solar cells prepared at such low temperatures. Secondly, this anatase TiO_2_ nanocrystalline films on SLG  substrate were employed as a sensor material to investigate selectivity for various volatile oragnic compunds (VOCs) such as ammonia, petrol, formaldehyde, ethanol, and acetone. The transient response, repeatability and response time for ammonia sensing were also determined as function of temperature and gas concentration. Finally, the stability of TiO_2_ film sensors with potential commercial viability is demonstrated.

## Results and Discussion

**Figure 1:**
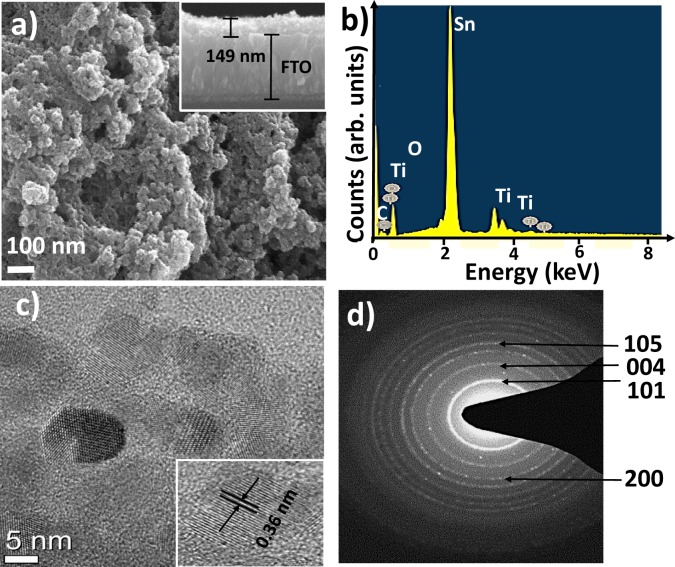
(**a**) FE-SEM plan-view image, (**b**) EDX pattern, (**c**) HR-TEM image, and (**d**) SAED pattern of SILAR-grown TiO_2_ film.

**Figure 2:**
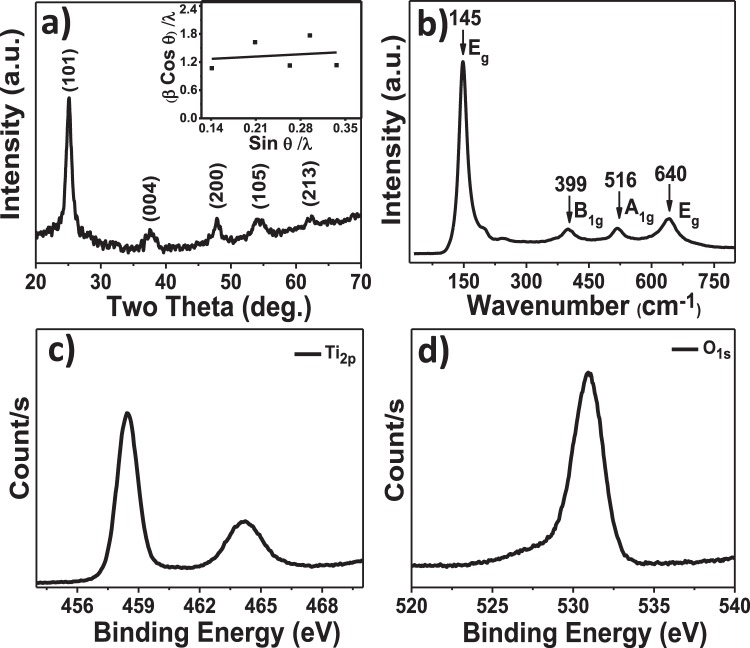
(**a**) XRD pattern (inset shows the W-H plot), (**b**) Raman spectrum of the TiO_2_ film, (**c**) Ti2_p_ and (**d**) O1_s_ core-level photoemission spectra.

Surface morphology and elemental analysis studies. The FE-SEM image shown in Fig. 1a demonstrates the spherical surface morphology of 15 L, optimized (discussed later), SILAR TiO_2_. Inset of Fig. 1a shows the cross-sectional view of TiO_2_ ETL which is relatively uniform of ~149 nm in thickness. The elemental surface composition analysis carried out by EDX (Fig. 1b) confirms stoichiometric quantities of Ti and O in for TiO_2_. The lattice parameters were measured from the HR-TEM images and the positions of the main diffraction peaks in the SAED patterns (Fig. 1c,d). The, 0.36 nm distance between two adjacent lattice planes was in accordance with the (101) reflection plane of anatase TiO_2_ (JCPDS card no. 21-1272)^21^. Being of lower surface energy, the (101) crystal face of TiO_2_ was more stable than the other facets^22^, and our HR-TEM images also demonstrated the strongest ring pattern for (101) plane than others in SAED spectrum. Moreover, the diffraction bright and sharp ring pattern confirms nanocrystalline nature of SILAR-mediated TiO_2_ film. The XRD pattern of SILAR-mediated TiO_2_ film on SLG substrate, which avoids FTO reflection contributions, is shown in Fig. 2a where well-defined reflections confirmed tetragonal crystal structure of anatase TiO_2_ (JCPDS card no. 21-1272)^23^. No other impurity/phase peak was noticed, indicating high phase purity of the product. The various structural parameters such as grain-size, dislocation densities, and texture coefficient values, determined from XRD pattern of TiO_2_ have tabulated in Table S1^†^ (Electronic Supporting Information, ESI). Furthermore, the dislocation density and texture coefficient values investigated from every diffraction peak are displayed in Table S1^†^. From Table S1^†^, the maximum texture coefficient of 2.011 was associated with (101) plane, which is also the preferred growth direction. The Williamson and Hall (W-H) plot (Fig. 2a inset) and the peak broadening method were utilized to estimate the average crystallite size as well as the micro-strain in the TiO_2_ particles. The positive values of slope and micro-strain (ε = 12.6 × 10^−3^) revealed an existence of tensile strain in TiO_2_ film. The average crystallite-size measured from the intercept value of W-H plot was 7 nm (±1 nm) which was in close agreement with the average value (7.2 nm) obtained from Scherrer’s formula and HR-TEM analysis i.e. 8 (±2) nm. The micro-strain was assigned to a relative change in interplanar distance which shifted diffraction peak positions, the difference in crystallite-size values (measured by W-H plot and Scherrer’s formula) and dislocation densities in TiO_2_ crystals^24^. The Raman spectrum of anatase TiO_2_ film (Fig. 2b) confirmed tetragonal space group D_4th_ (I41/amd) having six Raman active modes (1A_1g_ + 2B_1g_ + 3E_g_). The Raman peaks located at 143 (E_g_), 199 (Eg), 396 (B_1g_), 514 (A_1g_), and 636 cm^−1^ (E_g_) were assigned to the anatase TiO_2_^25^. In addition, as-presented in Fig. 2c,d, XPS analysis was used to confirm Ti^4+^ at TiO_2_ film surface. The XPS spectrum of TiO_2_ (Fig. S1^†^) showed Ti, O and C core level photoemissions, and there was no trace of any other impurity peak. The carbon peak at 284.8 eV was assigned to residual carbon in the sample, and adventitious carbon. The core-level emissions at binding energies of 458.9 and 464.6 eV are of Ti 2_p3/2_ and Ti 2_p1/2_, respectively^26^. The peak with a binding energy of 530 eV is from O1_s_, confirming presence of oxygen anion in the lattice (Ti-O-Ti)^27^_._ The BET-derived values of 121 m^2^/g specific surface-area and 6.54 nm average pore-size were confirm the mesoporous nature of the TiO_2_ material. A H1-type hysteresis loop supported for mesoporosity in anatase TiO_2_ film (obtained using scratched film powder)^28^. The adsorption-desorption isotherm and pore-size distribution plots for TiO_2_ are shown in Fig. S2^†^ and Table S2^†^. Since nanoparticles in TiO_2_ film were mesoporous in nature, with a high surface area and small pore-size, and uniformly distributed across the substrate surface, we investigated their potential for perovskite solar cell as well as efficient and selective gas sensor applications.

### Energy band positions

**Figure 3:**
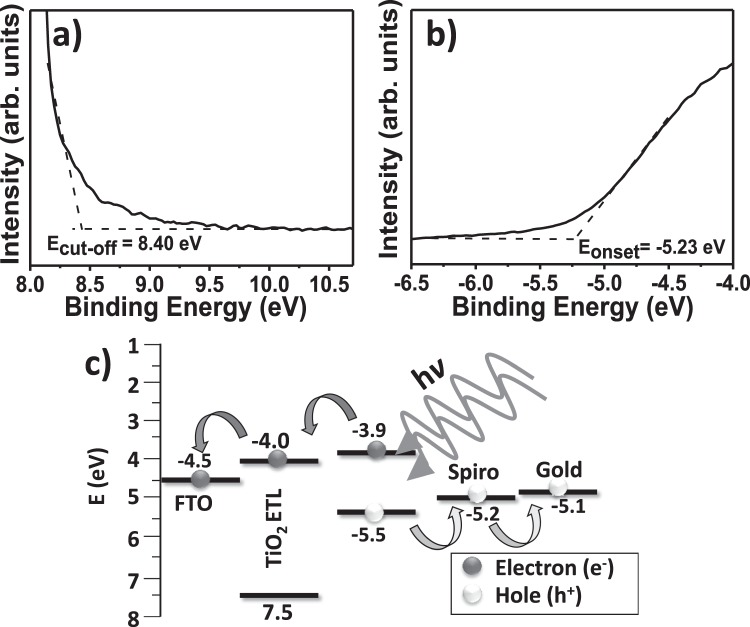
UPS spectra for different energy edge ranges: (**a**) cut-off region, (**b**) onset region of TiO_2_ film. (**c**) Schematic energy diagram of FTO/TiO_2_ ETL/perovskite/spiro-OMeTAD/gold solar cell device showing the energy level of each component calculated from UPS analysis.

To verify the energy level alignment, UPS measurements were performed, and the results are presented in Fig. 3a–c. The high binding energy cut-off (E_cut-off_) value of the TiO_2_ nanostructured film was determined to be 8.40 eV. The highest occupied molecular orbital (HOMO) energy onset value, the binding energy onset (E_onset_), relative to the Fermi level of Au (set at 0 eV), was measured to be −5.23 eV. The valence band minimum (VBM) was calculated according to eq. 1:

1 $${\rm{VBM}}={\rm{h}}{\rm{\nu }}-(\mathrm{Ecut} \mbox{-} \mathrm{off}-{\rm{Eonset}})$$

where, h is Planck’s constant and ν is the frequency of vibration of light, corresponding to −7.58 eV for TiO_2_. The optical band gap energy (Eg) of the TiO_2_ film was estimated using eq. 2:

2 $$({\rm{\alpha }}{\rm{h}}{\rm{\nu }})2={\rm{A}}({\rm{h}}{\rm{\nu }}-{\rm{Eg}})$$

where, A is the proportionality constant and α is the absorption coefficient. Fig. S3^†^ presents UV-vis spectrum of TiO_2_ film as an ETL where the E_g_ value was obtained by extrapolating the linear portion of the curve near the onset of the absorption edge to the energy axis (inset of Fig. S3^†^). The E_g_ value was determined to be 3.47 eV for the TiO_2_, which was close to the reported value^29^. Using the optical band gap, the conduction band minimum (CBM) energy level was also evaluated using eq. 3:

3 $${\rm{CBM}}={\rm{VBM}}+{\rm{Eg}}$$

The calculated CBM level for TiO_2_ was −4.0 eV. To obtain a clear understanding of the electron transfer pathway through the ETL, an energy band diagram of the FTO/TiO_2_ ETL/perovskite/spiro-OMeTAD/gold solar cell was proposed based on their appropriate band levels.

### Photovoltaic performance

**Figure 4:**
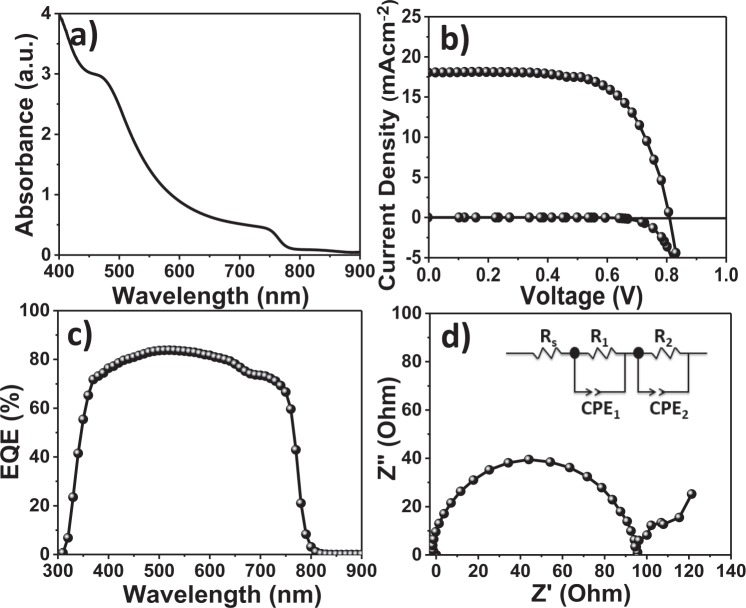
(**a**) UV-vis absorbance spectrum of deposited perovskite absorber layer FTO/TiO_2_ ETL/perovskite, (**b**) *J-V* curve data collected at 8.7 mV s^−1^ scan rate in reverse scan direction, (**c**) EQE spectrum for perovskite absorber layer, and (**d**) EIS spectra for FTO/TiO_2_ ETL/perovskite/spiro-OMeTAD/gold. All the data were collected using overnight aged cells.

**Figure 5:**
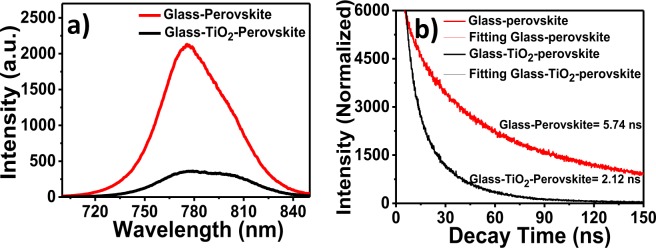
(**a**) Steady-state PL, and (**b)** Time resolved PL spectra of glass/TiO_2_ ETL/perovskite (excited with 325 nm He-Cd laser).

The UV-vis absorption spectrum of the perovskite absorber layer deposited on TiO_2_ ETL was obtained (Fig. 4a). The perovskite layer on TiO_2_ ETL demonstrates a wide absorbance in the visible region (centered at ~750 nm). The nanocrystallinity, smaller particle-size, mesoporous signature and higher surface area of the prepared SILAR-based anatase TiO_2_ film increased the overall perovskite adsorption, which was corroborated from the enhanced UV-Vis absorption. The performance of the perovskite solar cell was tested under illumination of simulated AM1.5 G simulated solar light (100 mW cm^−2^), and electronic parameters are listed in Table 1. The TiO_2_ ETL exhibited perovskite solar cell performance with an open circuit voltage (*V*_*OC*_) of 0.81 V, short-circuit current density (*J*_*SC*_) of 18.0 mA cm^−2^, and a fill factor (*ff* ) of 66.3% corresponding to a PCE (*ƞ*%) of 9.7% (Fig. 4b). To optimize the anatase TiO_2_ ETL thickness for best perovskite solar cell performance, the TiO_2_ ETL thickness was increased by changing SILAR cycles as 10, 15 and 20 L during deposition process, and detailed solar cell performances are shown in Fig. S4^†^ and Table S3^†^, respectively. For the 10 L SILAR deposition, the FTO/TiO_2_ ETL/perovskite/spiro-OMeTAD/gold device exhibited 8.5% PEC. Whereas, the device assembled with 20 L SILAR deposition had a PCE of 7.0%, and the best performance i.e. 9.7% was achieved for device fabricated for 15 L SILAR deposition at 8.7 mV s^−1^ scan rate in reverse scan direction. The comparative study of our TiO_2_ ETL with different synthesis methods for perovskite solar cell application was demonstrated in Table S4^†^^30–35^, signifying potentiality of proposed chemical method followed developed phase-pure TiO_2_ nanostructure in perovskite-based solar cells, which can be applied in developing other metal oxide nanostructured thin films too. To further verify the cell performance, EQE spectra of TiO_2_ ETL was obtained and presented in Fig. 4c. The TiO_2_ ETL device exhibited higher EQE value at 520 nm (~84.1%). Impedance spectroscopy in Fig. 4d (Nyquist plot) for the device conducted under 1 sun illumination at an applied bias voltage of 0.2 V, revealed two distinct semicircles (one in the high-frequency range and another in the low-frequency range) in the measured frequency range of 0.1 Hz to 100 kHz. The series resistance (R_S_) is related to the wiring and FTO substrate measured in the high-frequency region. R_1_ is the interface resistance of the counter electrode within the equivalent circuit representation, and CPE_1_ is its capacitance. In the Nyquist plot, the main arc is responsible for the TiO_2_ ETL/perovskite/spiro-OMeTAD interfaces as expressed by a combination of the interface recombination resistance (R_2_) and chemical capacitance (CPE_2_)^36^. The PL measurement confirms the photo-induced charge transfer and charge recombination loss in optolectrochemical devices^37^. Figure 5a presents the PL spectra of the glass/perovskite and glass/TiO_2_/perovskite layer configurations. The PL intensity of the glass/TiO_2_/perovskite film was less than that of the glass/perovskite film, indicating that the charge transfer effectively occurs prior to non-radiative carrier recombination at the interface, and this improved the electron extraction rate from the perovskite absorber layer. The TRPL results of the perovskite films deposited on glass/perovskite and glass/TiO_2_/perovskite are shown in Fig. 5b. The perovskite film deposited on glass/perovskite has an estimated charge carrier lifetime of 5.74 ns where, in contrast, the charge carrier lifetime of the perovskite film deposited on glass/TiO_2_/perovskite was ~37% times shorter (τ = 2.12 ns), evidencing a faster electron transfer process in the latter case. The decrease in the PL intensity supported the enhancement in PCE for the glass/TiO_2_/perovskite-based device counterpart. To verify the reproducibility of the perovskite solar cells, 10 devices at each TiO_2_ ETL were fabricated. Fig. S5^†^ presents the average values of the photovoltaic device parameters. The perovskite solar cells assembled from SILAR-mediated TiO_2_ ETL devices exhibited good reproducibility of *J*_*sc*_, *V*_*oc*_, *ff*, and PCE^37^. To confirm the hysteresis behavior of TiO_2_ ETL, different scan rates (i.e. as high as 520 mV s^−1^ and as slow as 8.7 mV s^−1^) and scan directions (reverse scan: black solid symbol and forward scan: black open symbol) were operated on the TiO_2_ ETL-based perovskite solar cell (Fig. S6a–c^†^) whose data is summarized in Table S7^†^. A very little hysteresis was obtained at slow scan rate i.e. 8.7 mV s^−1^ as there was a light difference between the PCE values obtained during the reverse (9.7%) and forward (9.1%) scans, suggesting chemical stability and mechanical robustness of developed ETL layer (Fig. S6c^†^).

**Table 1:** Summary of photovoltaic performance of TiO_2_ ETL/perovskite/spiro-OMeTAD/gold device fabricated with 10 SILAR cycles.

ETL	*Jsc* (mA/cm^2^)	*V*_*oc*_ (V)	*ff* (%)	*η* (%)
TiO_2_	18.0	0.81	66.3	9.7
Average	15.5	0.80	65.5	7.5
Std. Dev.	2.53	0.01	0.86	2.2

Efficiency data were obtained by averaging the response from 10 devices under AM 1.5 illumination (100 mW cm^−2^).

### Gas sensor activities

**Figure 6:**
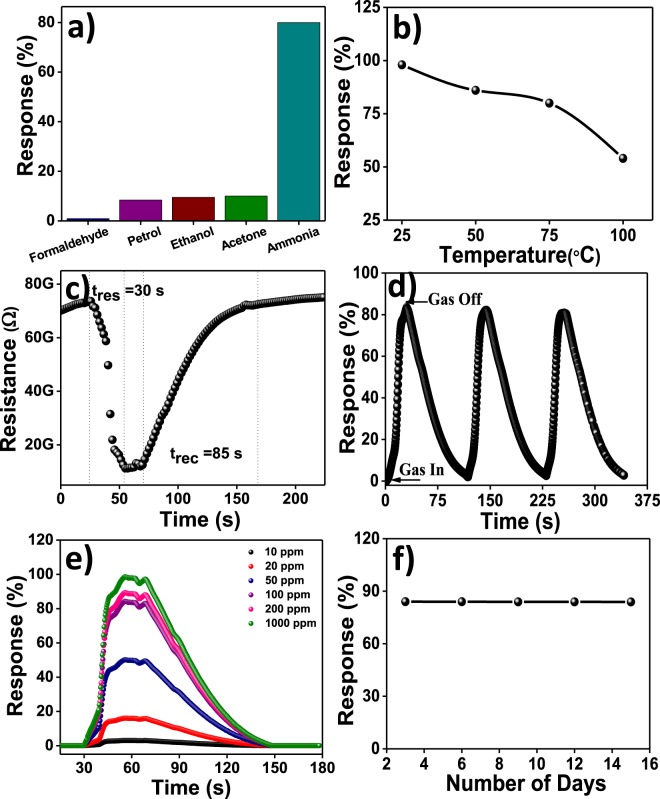
SILAR-mediated anatase TiO_2_ film sensor; (**a**) selectivity of towards various VOCs gases at room temperature (300 K), (**b**) operating temperature optimization, (**c**) transient response and recovery curves, (**d**) repeatability towards ammonia at 100 ppm concentration, (**e**) response for various ammonia concertation levels (10–1000 ppm), and (**f**) long-term stability test towards ammonia.

It is well-known that the gas sensing mechanism is based on the alteration in the resistance of the sensing material, which is influenced by the adsorption-desorption process of target gas molecules *via* charge transfer processes. The resistance of the TiO_2_ film was stabilized before exposure to various VOCs *viz*. ammonia, petrol, formaldehyde, ethanol, and acetone to a maximum of 100 ppm. Sensitivity, operating temperature and transient gas response study of the anatase TiO_2_ nanocrystalline SLG  substrate supported film (25 L) was envisaged for sensitivity study for various gases as for gas sensor thick film is essential. Above this condition, films started peeling off from the substrate surface due to its weak mechanical adhesion. The sensitivity study of TiO_2_ sensor for 100 ppm gas concentration at room-temperature is shown in Fig. 6a. The TiO_2_ nanocrystalline film showed better sensitivity (80%) towards ammonia over other target gases. The ammonia gas response of the TiO_2_ sensor as function of operating temperature was studied (Fig. 6b). The response was recorded maximum at room-temperature (300 K) whereas, it decreased with at higher temperatures. The maximum ammonia response at room-temperature could be because of high number of ambient oxygens as well as target gas molecules adsorption on the TiO_2_ sensor surface; whereas decreased adsorption process for higher operating temperatures deteriorated the ammonia detection response^38^. The transient ammonia response of anatase TiO_2_ nanocrystalline film sensor is shown in Fig. 6c. As the ammonia exposed in testing chamber, the diffusing ammonia molecules adsorb on the TiO_2_ film sensor surface over time. After specific time, sensor reached to its equilibrium (saturation level), where ammonia response remained constant. Furthermore, the sensing system was opened to external atmosphere after saturation. The ammonia molecules were desorbed and hence, the ammonia response decreased. With time, TiO_2_ sensor achieved its original state of resistance. The response and recovery time values of TiO_2_ film sensor for ammonia sensing were 30 and 85 s, respectively, signifying the use of TiO_2_ material as potential room-temperature ammonia sensor. The dynamic repeatability study of TiO_2_ sensor was studied and is shown in Fig. 6d. The 100 ppm ammonia gas was tested for several times at room-temperature in order to observe sensor. It was observed that TiO_2_ sensor shows approximately same ammonia response over a few cycles, signifying an effective and repeatable contribution of sensor adsorption/desorption sites. The ammonia response of the TiO_2_ sensor was studied for various concentrations at the room-temperature (Fig. 6e). The lowest ammonia concentration detected response at 10 ppm was 2.5%. The ammonia response increased with concentration. The ammonia response of TiO_2_ film sensor increased until the sensor surface available for adsorption of target ammonia molecules. The highest ammonia response for 1000 ppm concentration was 98%. The comparison of reported sensor performances of different materials used for ammonia detection is provided in theTable S5^†^ and also the sensor performances of previously reported TiO_2_ sensor materials for different gases are given in the Table S6^†^^39–48^. For higher ammonia concentration above 1000 ppm, the gas response gets was saturated, which we assume is due to maximal coverage for the TiO_2_ surface by ammonia molecules to the point where limited resistance changes are detected. The stability plot of TiO_2_ film for ammonia sensing is shown in Fig. 6f. The response stability of TiO_2_ sensor towards 100 ppm concentrations of ammonia was studied for 15 days and observed to be approximately constant.

### Sensing mechanism

The gas-sensing mechanism of sensor involves the adsorption of gas molecules on the metal oxide film surface and charge redistribution between the surface and the adsorbed molecules, which eventually produces changes in the electronic structure and conductivity of sensor material^49^. The ammonia sensing mechanism on TiO_2_ film sensor followed two stages i.e. oxidation and reduction. In oxidation process, air oxygen species oxidizes the TiO_2_ sensor surface by capturing conduction electron offering depletion region underneath TiO_2_ sensor surface and consequently conductional channel shrinks^50^. The width of depletion region formed depends on number of air oxygen species adsorbed and number of conduction electrons available in TiO_2_ sensor at the measurement temperature^51^. These interconnected space-charge depleted TiO_2_ nanoparticles within the sensor film offer additional resistance to that of the grain boundary resistance, because of an increased Schottky barrier potential (qV_b_) in air^52^. In the second stage, ammonia gas absorption reduces the TiO_2_ sensor surface by donating electrons *via* chemisorbed anion species according to^38^:

4 $${{\rm{O}}}_{2({\rm{air}})}\to {{\rm{O}}}_{2({\rm{ads}})}$$

5 $${{\rm{O}}}_{2({\rm{ads}})}+{{\rm{e}}}^{-}\to {{\rm{O}}}_{2({\rm{ads}})}^{-}$$

6 $$4{{\rm{NH}}}_{3({\rm{gas}})}+5{{\rm{O}}}_{2({\rm{ads}})}^{-}\to 4{\rm{NO}}+6{{\rm{H}}}_{2}{\rm{O}}+5{{\rm{e}}}^{-}$$

In this reduction process, the overall depletion width of TiO_2_ reduces by gaining electrons from oxygen ion species and increasing overall conductivity and reducing the Schottky barrier height. Consequently, the inter-particle conduction pathway width is increased rendering the entire film more conductive.

## Conclusion

In summary, mesoporous anatase TiO_2_ nanocrystalline films were directly growth on FTO and soda-lime glass substrates by using a simple SILAR method at low-temperature i.e. 90 °C. The morphological, structural, optical and electrical properties of TiO_2_ nanocrystalline films are consistent across the substrate allowing ETL layers to be grown for perovskite solar cells. The best performing perovskite solar cell was constructed using a 15-layer SILAR grown TiO_2_ ETL achieved a PCE of ~9.7%, with *J*_*SC*_ of 18.05 mA/cm^2^, *V*_*OC*_ of 0.81 V and *ff* of 66.3%. The considerable 84% room-temperature sensor response of anatase TiO_2_ film sensor towards ammonia among various volatile gases demonstrates the film’s capability for resistance-based sensing. A wide range of gas concentrations detected by TiO_2_ film sensor at room-temperature confirmed the 2.5% response as lowest ammonia detection limit at 10 ppm whereas, at 1000 ppm it was 98%. The fast response and recovery time values of present sensor for ammonia sensing were recorded as 30 s and 85 s, and nearly fifteen days stability, respectively. As-prepared anatase TiO_2_ nanocrystalline film-based ammonia sensor demonstrated good repeatability, high selectivity, stability, and fast transition times, signifying its potentiality as room-temperature VOCs based gas sensor. Furthermore, the use of this novel process may in future find great potential in large-scale fabrication of perovskite solar cell and gas sensor models in practical applications.

### Experimental Section

All the chemicals used as reactants were of analytical grade, and used without any further purification. All the other materials were purchased from Sigma-Aldrich, including titanium (IV) chloride (TiCl_4_), potassium persulfate (K_2_S_2_O_8_, 99%), lead iodide (PbI2, 99.9%), methylammonium iodide (MAI, Dyesol), dimethyl sulfoxide (DMSO, 99.9%), N-N-dimethylformamide (DMF, 99.8%), isopropanol, and chlorobenzene (99.8%) were used in the as-received condition. In addition, 2, 2′, 7, 7′-tetrakis (N, N-di-p-methoxyphenyl-amine) 9, 9′-spirobifluorene (spiro-MeOTAD) was obtained from Borun Chemicals (98%, Ningbo, China). All VOCs were obtained SD FineChem Limited, Mumbai, for gas sensing purpose. The laser-patterned FTO (15 Ω sq^−1^) and soda-lime glass substrates (Borosil) were cleaned with diluted detergent solution, ultra-sonicated for 10 min with deionized water, acetone, and ethanol, and dried with clean dry nitrogen air. These substrates were ultrasonically cleaned for 30 min prior to TiO_2_ deposition.

### Preparation of SILAR TiO_2_

Synthesis of anatase TiO_2_ nanocrystalline films on two substrates viz. FTO and glass was carried out by low-temperature SILAR chemical deposition. Briefly, 0.1 M TiCl_4_ was prepared in 50 ml de-ionized water as the Ti4+ source (eq. 1). In another beaker, 0.1 M (50 ml) K_2_S_2_O_8_ was prepared (eq. 2) and kept at 90 °C constant temperature and used as sulphate/oxide source (eq. 3). The substrate (either FTO or glass) was dipped in 0.1 M TiCl_4_ solution for 20 s and then the same substrate was dipped in de-ionized water for 10 s to remove remaining loosely bonded ions. A similar procedure was performed in 0.1 M K_2_S_2_O8 solution at 90 °C using the same substrate, representing one cycle (eq. 4). After about ten such cycles, a whitish film started to appear on both substrates. The dipping cycles were continued until twenty on FTO substrate and twenty-five on the glass substrate were coated, to form uniform and relatively thick whitish TiO_2_ films. The deposited films were rinsed with de-ionized water and air calcined at 450 °C temperature for 1 h to obtain anatase TiO_2_ nanocrystalline films (eq. 5), whose chemical reaction mechanism is proposed below which further were characterized and employed for perovskite solar cell and chemical gas sensing applications.

7 $${{\rm{TiCl}}}_{4}\to {{\rm{Ti}}}^{+4}+4{{\rm{Cl}}}^{-}$$

8 $${{\rm{K}}}_{2}{{\rm{S}}}_{2}{{\rm{O}}}_{8}\to 2{{\rm{K}}}^{+}+{{\rm{S}}}_{2}{{{\rm{O}}}_{8}}^{--}$$

9 $${{\rm{S}}}_{2}{{{\rm{O}}}_{8}}^{2-}\,\mathop{\longrightarrow }\limits^{{\rm{\Delta }}90\,{}^{\circ }{\rm{C}}}\,2{{{\rm{SO}}}_{4}}^{--}$$

10 $${{\rm{Ti}}}^{+4}+{{{\rm{SO}}}_{4}}^{2-}\,\mathop{\longrightarrow }\limits^{{\rm{\Delta }}90\,{}^{\circ }{\rm{C}}}\,{\rm{Ti}}{({{\rm{SO}}}_{4})}_{4}$$

11 $${\rm{Ti}}{({{\rm{SO}}}_{4})}_{4}\,\mathop{\longrightarrow }\limits^{{\rm{\Delta }}450\,{}^{\circ }{\rm{C}}}\,{{\rm{TiO}}}_{2}$$

### TiO_2_ ETL layer-perovskite device fabrication

The PbI_2_ was dissolved in DMF at a concentration of 462 mg per ml under stirring at 70 °C. The solution was kept at 70 °C during the whole deposition procedure. The PbI_2_ precursor was spin-coated on SILAR-based anatase TiO_2_ nanocrystalline ETL at 4000 rpm for 30 s and dried at 70 °C for 10 min. After cooling to room-temperature, the films were then dipped in a solution of MAI in isopropanol (15 mg per ml) for 20 s, rinsed with isopropanol, and dried by nitrogen gas. A volume of 60 μl spiro-OMeTAD solution was spin-coated on the perovskite/TiO_2_ layer at 3000 rpm for 30 s. The solution was prepared by dissolving 72 mg spiro-OMeTAD in 1 ml of chlorobenzene, to which 28.8 μl of 4-tert-butyl pyridine and 14.4 μl of lithium bis(trifluoromethanesulfonyl)imide (Li-TFSI) solution (520 mg Li-TFSI in 1 ml of acetonitrile, Sigma-Aldrich, 99.8%) were added. Finally, 70 nm of gold was deposited at ~10^−6^ bar *via* thermal evaporation on the spiro-OMeTAD coated film for electrical contacts.

### Gas sensing measurement details

For the gas response measurement, changes in electrical resistance of TiO_2_ film sensors during the interaction of target gases were recorded and the response (S) of sensor was calculated according to:

12 $${\rm{S}}=\frac{{{\rm{R}}}_{{\rm{a}}}-{{\rm{R}}}_{{\rm{g}}}}{{{\rm{R}}}_{{\rm{a}}}\,}\times 100$$

where, R_a_ and R_g_ are the resistances of TiO_2_ film sensor in presence of air and target gas, respectively. The desired concentration of target gas was obtained by the static liquid gas distribution method, which was calculated by the following formula^27^:

13 $$C\,({\rm{ppm}})=\frac{22.4{\rm{\rho }}\mathrm{TV}\text{'}}{273{\rm{MV}}}\times 1000$$

where, C (ppm) is the desired target gas concentration; ρ (58.08 g mol^−1^) is the density of the liquid, V′ is the volume of liquid acetone (µL), T (298 K) is testing temperature, M is the molecular weight of acetone (0.788 g cm^−3^), and V is the volume of testing chamber (0.250 L).

### Characterization tools

Surface morphology and elemental composition of TiO_2_ films were analyzed using field-emission scanning electron microscopy (FE-SEM, JSM-7001F, JEOL Ltd., Tokyo, Japan) digital images and energy dispersive X-ray (EDX) pattern, respectively. The high resolution transmission electron microscopy (HR-TEM, Titan 80–300, FEI, Hillsboro, OR, USA) and selected area electron diffraction (SAED) images were recorded using a FEI TECNAI G2 20 S-TWIN equipped with a LaB6 cathode and a GATAN MS794 PCCD camera. These micrographs were obtained at an acceleration voltage of 200 kV. The powder of TiO_2_ film sample was suspended in ethanolic solution separately and dropped onto a Formvar/carbon, 200 mesh copper grids before HR-TEM measurements. The phase of TiO_2_ film was confirmed from X-ray diffraction (XRD) pattern (XRD-6000, Shimadzu, Japan) with Cu-Kα radiation (λ = 0.1542 nm). Diffraction pattern was recorded from 20° to 70° (2θ) with a 2° min^−1^ scan speed. The phase analysis was additionally performed using a Raman scattering spectrum scanned on Renishaw Invia Raman Microscope. The laser radiation (λ = 532 nm) was focused on the TiO_2_ film surface. The spectrum was measured from 100 to 1000 cm^−1^. The surface-sensitive quantitative spectroscopic technique of X-ray photoelectron spectroscopy (XPS) and ultraviolet photoelectron spectroscopy (UPS) spectra of the TiO_2_ nanostructures were acquired with PHI 5000 Versa Probe (Ulvac-PHI) under high vacuum conditions (6.8 × 10^−8^ pa) using a monochromatic Al Kα X-ray source (1486.6 eV). The data was collected from a spot size of 100 × 100 μm^2^. The carbon 1 s photoemission line (284.6 eV) was used for internal calibration as a reference. The optical properties of the TiO_2_ nanostructured films were measured at room-temperature using a UV-vis spectrophotometer (V-530, Jasco). The photovoltaic performances and current-voltage (*JV*) characteristics of perovskite solar cells, for various synthesis conditions, were determined using a solar simulator (Sol3A Class AAA, Oriel Instruments) and a Keithley 2400 source measurement unit. The AM1.5 G simulated solar light (100 mW cm^−2^) light intensity level was calibrated using a standard Si reference cell certified by the Newport Corporation. All the devices were measured in a light-tight sample holder, with an active area of 0.06 cm^2^ for each cell fixed using an aperture mask. The external quantum efficiency (EQE) was recorded using a quantum efficiency measurement system (QEX10, PV Measurements, Inc.) as a function of wavelength from 300 to 850 nm. The steady-state photoluminescence (PL) and time-resolved photoluminescence (TRPL) spectroscopy spectra of perovskite-coated on both the glass and glass/TiO_2_ were also performed using a micro-photoluminescence measurement system (IK350IR-G, 325-nm He-Cd laser). The gas sensing properties of TiO_2_ films gown directly on soda-lime glass were measured in a stainless-steel cylindrical chamber with 250 ml volume capacity. Resistivity in air and target gases were measured using a two-electrode arrangement, with silver paint metal contacts of 10 mm × 10 mm area, using a Keithley 6514 electrometer as a function of time and gas concertation. In order to control temperature, a small heater equipped with a thermocouple was used.

## Electronic supplementary material

Supplementary data

## References

[1] Wang QH, Kalantar-zadeh K, Coleman KAJN, Strano MS (2012). Electronics and optoelectronics of two-dimensional transition metal dichalcogenides. Nat. Nanotechnol..

[2] Manzeli S, Ovchinnikov D, Pasquier D, Yazyev OV, Kis A (2017). 2D transition metal dichalcogenides. Nature Reviews Materials.

[3] Tack LW, Azam MA, Seman RNAR (2017). Structural and Electronic Properties of Transition-Metal Oxides Attached to a Single-Walled CNT as a Lithium-Ion Battery Electrode: A First-Principles Study. J. Phys. Chem. A.

[4] Zazpe R (2017). Atomic layer deposition Al_2_O_3_ coatings significantly improve thermal, chemical, and mechanical stability of anodic TiO_2_ nanotube layers. Langmuir.

[5] Ke W (2016). TiO_2_–ZnS cascade electron transport layer for efficient formamidinium tin iodide perovskite solar cells. J. Americ. Chem. Soc..

[6] Chen D, Huang F, Cheng YB, Caruso RA (2009). Mesoporous anatase TiO_2_ beads with high surface areas and controllable pore sizes: a superior candidate for high-performance dye-sensitized solar cells. Adv. Mater..

[7] Zhang H, Banfield JF (1998). Stability of nanosized TiO_2_ particles. J. Mater. Chem.

[8] Gopal M, Chan WJM, De Jonghe IC (1997). Room temperature synthesis of crystalline metal oxides. J. Mater. Sci..

[9] Feng X (2008). Vertically aligned single crystal Ti_O2_ nanowire arrays grown directly on transparent conducting oxide coated glass: synthesis details and applications. Nano letters.

[10] Roy P, Kim D, Lee K, Spiecker E, Schmuki P (2010). TiO_2_ nanotubes and their application in dye-sensitized solar cells. Nanoscale.

[11] Liu B, Aydil ES (2009). Growth of oriented single-crystalline rutile TiO_2_ nanorods on transparent conducting substrates for dye-sensitized solar cells. J. Americ. Chem. Soc..

[12] Fang B (2014). Large-scale synthesis of TiO_2_ microspheres with hierarchical nanostructure for highly efficient photodriven reduction of CO_2_ to CH_4_. ACS appl. Mater. Interface..

[13] Raut NC (2011). Effect of temperature on the growth of TiO_2_ thin films synthesized by spray pyrolysis: structural, compositional and optical properties. Mater. Res. Bullet..

[14] Kaper H, Sallard S, Djerdj I, Antonietti M, Smarsly BM (2010). Toward a Low-Temperature Sol^−^ Gel Synthesis of TiO_2_ (B) Using Mixtures of Surfactants and Ionic Liquids. Chem. Mater..

[15] Cao FF, Xin S, Guo YG, Wan LJ (2011). Wet chemical synthesis of Cu/TiO_2_ nanocomposites with integrated nano-current-collectors as high-rate anode materials in lithium-ion batteries. Phys. Chem. Chem. Phys..

[16] Shao Z, Zhu W, Li Z, Yang Q, Wang G (2012). One-step fabrication of CdS nanoparticle-sensitized TiO2 nanotube arrays via electrodeposition. J. Phys. Chem. C.

[17] Senthilkumar V, Jayachandran M, Sanjeeviraja C (2010). Preparation of anatase TiO_2_ thin films for dye-sensitized solar cells by DC reactive magnetron sputtering technique. Thin Solid Films.

[18] Shi J, Wang X (2011). Growth of rutile titanium dioxide nanowires by pulsed chemical vapor deposition. Crystal Growth & Design.

[19] Pathan HM, Min SK, Desai JD, Jung KD, Joo OS (2006). Preparation and characterization of titanium dioxide thin films by SILAR method. Mater. Chem. Phys..

[20] Burschka J (2013). Sequential Deposition As a Route to High-Performance Perovskite-Sensitized Solar Cells. Nature.

[21] Zhang Z (2013). Hierarchical assembly of ultrathin hexagonal SnS_2_ nanosheets onto electrospun TiO_2_ nanofibers: enhanced photocatalytic activity based on photoinduced interfacial charge transfer. Nanoscale.

[22] Tachikawa T, Yamashita S, Majima T (2011). Evidence for crystal-face-dependent TiO_2_ photocatalysis from single-molecule imaging and kinetic analysis. J. Americ. Chem. Soc..

[23] Shaikh SF, Mane RS, Min BK, Hwang YJ, Joo OS (2016). D-sorbitol-induced phase control of TiO_2_ nanoparticles and its application for dye-sensitized solar cells. Scientific reports.

[24] Patil UM, Gurav KV, Joo OS, Lokhande CD (2009). Synthesis of photosensitive nanograined TiO2 thin films by SILAR method. Journal of Alloys and Compounds.

[25] Chai B (2013). Synthesis of C60-decorated SWCNTs (C 60-d-CNTs) and its TiO_2_-based nanocomposite with enhanced photocatalytic activity for hydrogen production. Dalt. Trans..

[26] Ali SMM, Sandhya KY (2016). One step solvothermal synthesis of ultra-fine N-doped TiO_2_ with enhanced visible light catalytic properties. RSC Adv..

[27] Preethi LK, Antony RP, Mathews T, Walczak L, Chinnakonda S (2017). A Study on Doped Heterojunctions in TiO_2_ Nanotubes: An Efficient Photocatalyst for Solar Water Splitting. Nat. Sci. Reports.

[28] Xiaojia L, Mingming Z, Yang W (2017). Soft-Template Synthesis of Mesoporous Anatase TiO_2_ Nanospheres and Its Enhanced Photoactivity. Molecules.

[29] Chekini M, Mohammadizadeh MR, Allaei SV (2011). Photocatalytic and superhydrophilicity properties of N-doped TiO_2_ nanothin films. Appl. Surf. Sci..

[30] Niu G (2014). Study on the stability of CH_3_NH_3_PbI_3_ films and the effect of post-modification by aluminum oxide in all-solid-state hybrid solar cells. J. Mater. Chem. A.

[31] Shaikh SF (2016). La_2_O_3_ interface modification of mesoporous TiO_2_ nanostructures enabling highly efficient perovskite solar cells. J. Mater. Chem. A.

[32] Lee JW (2014). Rutile TiO_2_-based perovskite solar cells. J. Mater. Chem. A.

[33] Kim HS (2013). High efficiency solid-state sensitized solar cell-based on submicrometer rutile TiO_2_ nanorod and CH_3_NH_3_PbI_3_ perovskite sensitizer. Nano letters.

[34] Mali SS (2015). Ultrathin atomic layer deposited TiO_2_ for surface passivation of hydrothermally grown 1D TiO_2_ nanorod arrays for efficient solid-state perovskite solar cells. Chem. Mater..

[35] Su TS, Hsieh TY, Hong CY, Wei TC (2015). Electrodeposited ultrathin TiO_2_ blocking layers for efficient perovskite solar cells. Scientific reports.

[36] Liang C (2017). Chemical bath deposited rutile TiO_2_ compact layer toward efficient planar heterojunction perovskite solar cells. Appl. Surf. Sci..

[37] Huang L (2017). Efficient and hysteresis-less pseudo-planar heterojunction perovskite solar cells fabricated by a facile and solution-saving one-step dip-coating method. Organ. Electron..

[38] Ghule BG (2017). Natural Carbonized Sugar as a Low-Temperature Ammonia Sensor Material: Experimental, Theoretical, and Computational Studies. ACS appl. mater. interface..

[39] Dasari BS (2015). Room temperature single walled carbon nanotubes (SWCNT) chemiresistive ammonia gas sensor. Sens.Trans..

[40] Sharma S, Hussain S, Singh S, Islam SS (2014). MWCNT-conducting polymer composite based ammonia gas sensors: A new approach for complete recovery process. Sens. Act. B: Chem..

[41] Nguyen LQ, Phan PQ, Duong HN, Nguyen CD, Nguyen LH (2013). Enhancement of NH_3_ gas sensitivity at room temperature by carbon nanotube-based sensor coated with Co nanoparticles. Sensors.

[42] Wu Z (2013). Enhanced sensitivity of ammonia sensor using graphene/polyaniline nanocomposite. Sens. Act. B: Chemical.

[43] Travlou NA, Bandosz TJ (2017). Nanoporous carbon-composites as gas sensors: Importance of the specific adsorption forces for ammonia sensing mechanism. Carbon.

[44] Lin S, Li D, Wu J, Li X, Akbar SA (2011). A selective room temperature formaldehyde gas sensor using TiO_2_ nanotube arrays. Sens. Act. B: Chemical.

[45] Lu HF (2008). Amorphous TiO_2_ nanotube arrays for low-temperature oxygen sensors. Nanotech..

[46] Dhawale DS (2008). Room temperature liquefied petroleum gas (LPG) sensor based on p-polyaniline/n-TiO_2_ heterojunction. Sens. Act. B: Chem..

[47] Şennik E, Colak Z, Kılınç N, Öztürk ZZ (2010). Synthesis of highly-ordered TiO_2_ nanotubes for a hydrogen sensor. Internat. J. Hydro. Ener..

[48] Perillo PM, Rodriguez DF (2014). A room temperature chloroform sensor using TiO_2_ nanotubes. Sens. Act. B: Chem..

[49] Yang F, Guo Z (2016). Engineering NiO sensitive materials and its ultra-selective detection of benzaldehyde. J. colloid. interfac. sci..

[50] Hu J (2017). Synthesis and characterization of flower-like MoO_3_/In_2_O_3_ microstructures for highly sensitive ethanol detection. RSC Adv..

[51] Wang C, Yin L, Zhang L, Xiang D, Gao R (2010). Metal oxide gas sensors: sensitivity and influencing factors. Sensors.

[52] Zhou X (2015). Highly enhanced sensing properties for ZnO nanoparticle-decorated round-edged α-Fe2O3 hexahedrons. ACS appl. mater. Interf..

