# Author Correction: Low-Temperature Ionic Layer Adsorption and Reaction Grown Anatase TiO_2_ Nanocrystalline Films for Efficient Perovskite Solar Cell and Gas Sensor Applications

**DOI:** 10.1038/s41598-019-43987-w

**Published:** 2019-06-05

**Authors:** Shoyebmohamad F. Shaikh, Balaji G. Ghule, Umesh T. Nakate, Pritamkumar V. Shinde, Satish U. Ekar, Colm O’Dwyer, Kwang Ho Kim, Rajaram S. Mane

**Affiliations:** 1 School of Physical Sciences, Swami Ramanand Teerth Marathwada University, Nanded, 431 606 India; 2 School of Chemistry, University College of Cork, Cork, T12 YN 60 Ireland; 3 Micro-Nano Systems Centre, Tyndall National Institute, Lee Maltings, Cork, T12 R5CP Ireland; 4 Environmental Research Institute, University College Cork, Lee Road, Cork, T23 XE10 Ireland; 5 Hybrid Material Solution National Core Research Center, Pusan National University, Busan, 600-735 Republic of Korea

Correction to: *Scientific Reports* 10.1038/s41598-018-29363-0, published online 20 July 2018

This Article contains typographical errors in the Acknowledgements section.

“Authors KHK and RSM are indebted to Global Frontier Program through the Global Frontier Hybrid Interface Materials (GFHIM) of the National Research Foundation of Korea (NRF) funded by the Ministry of Science and ICT (2013M3A6B1078869) for financial support.”

should read:

“Authors KHK and RSM are indebted to Global Frontier Program through the Global Frontier Hybrid Interface Materials (GFHIM) of the National Research Foundation of Korea (NRF) funded by the Ministry of Science and ICT (2013M3A6B1078874) for financial support.”

