# Supplementary material for: Low-Temperature Ionic Layer Adsorption and Reaction Grown Anatase TiO2 Nanocrystalline Films for Efficient Perovskite Solar Cell and Gas Sensor Applications
Source: Sci Rep. 2018 Jul 20;8:11016. doi: 10.1038/s41598-018-29363-0 (PMC6054626; doi:10.1038/s41598-018-29363-0)
Supplement: Supplementary file 1 — Supplementary data [file 41598_2018_29363_MOESM1_ESM.docx]

**Supplementary Information**

**Low-Temperature Ionic Layer Adsorption and Reaction Grown Anatase TiO_2_ Nanocrystalline Films for Efficient Perovskite Solar Cell and Gas Sensor Applications**

Shoyebmohamad F. Shaikh,^1^ Balaji G. Ghule^1^ Umesh T. Nakate ^1^ Pritamkumar V. Shinde,^1^ Satish U. Ekar,^1^ Colm O’Dwyer ^2*^ Kwang Ho Kim^3*^nd Rajaram S. Mane^1*^

*^1^*School of Physical Sciences, Swami Ramanand Teerth Marathwada University, Nanded, 431 606, India

*^2^*School of Chemistry, University College of Cork, Cork T12 YN 60, Ireland.

*^2^*Micro-Nano Systems Centre, Tyndall National Institute, Lee Maltings, Cork T12 R5CP, Ireland.

*^2^*Environmental Research Institute, University College Cork, Lee Road, Cork T23 XE10, Ireland.

*^3^*National Core Research for Hybrid Materials Solution, Pusan National University, Busan 600-735, Republic of Korea. **^^**

**Fig. S1** Binding energy survey spectra of TiO_2_ nanostructure film.

**Fig. S2** BET adsorption-desorption isotherm for TiO_2_ nanostructure powder.

**Fig. S3** UV-vis absorbance spectra of TiO_2_ ETL before the deposition of perovskite. The inset Tauc plot shows the band gap position of the TiO_2_ ETL.

**Fig. S4** *JV* characteristics of the FTO/TiO_2_ ETL/perovskite/spiro-OMeTAD /gold device under illumination of simulated AM1.5G light (100 mW cm^-2^). The TiO_2_ ETL varied using different SILAR Cycle layers times of 10, 15 and 20 Cycle. The scan rate was constant at 8.6 mV s^-1^ for all the measurements**.**

**Fig. S5** Device parameter statistics demonstrating the consistency in the performance of 10 devices: (a) *J_SC_,* (b) *V_OC_*, (c) *ff,* and (d) PCE of FTO/TiO_2_/perovskite/spiro-OMeTAD/gold. The 25^th^, 50^th^, and 75^th^ percentiles of the device parameters are represented with three horizontal lines in the box. The average and maximum/minimum values are denoted by ■ and ×, respectively.

**Fig. S6** *JV* characteristics of forward scan and reverse scan for FTO/TiO_2_ ETL/perovskite /spiro-OMeTAD/ gold device under illumination of simulated AM1.5G light (100 mW cm^-2^) for scan rates of (a) 520, (b) 52, and (c) 8.7 mV s^-1^.

**Table S1** Structural parameters of TiO_2_ nanostructures film.

| **Molar concentration** | **Standard values/Formula** | **(hkl)** | **Estimated**  **Values (nm)** |
| --- | --- | --- | --- |
| Crystallite size (D) (nm) Scherrer’s formula | $D=\frac{K.\lambda}{\beta.\cos\theta}$ | 101  004  200  105 | 8  6  7  8 |
| Average crystallite size | | | 7.2 |
| d – spacing (Å)  JCPDS -  Card no. 21-1272 | 3.5168  2.3782  1.8925  1.6999 | 101  004  200  105 | 3.5408  2.3948  1.8994  1.6789 |
| Diffraction peak 2θ (^0^) | 25.281  37.800  48.049  53.890 | 101  004  200  105 | 25.119  37.510  47.830  53.990 |
| Dislocation density (lines/m^2^) X 10^15^ | δ = 1/D^2^ | 101  004  200  105 | 15.625  27.777  15.625  40.000 |
| Texture coefficient TC | ${TC}_{hkl}=\frac{\frac{I_{hkl}}{I_{0}}}{\frac{1}{N}\sum\frac{I_{hkl}}{I_{0}}}$ | 101  004  200  105 | 2.011  1.373  0.492  1.124 |
| Micro strain (ε) | $\frac{cos\theta}{\lambda}$=$\frac{1}{D}+\frac{sin\theta}{\lambda}$ |  | 12.6 x 10^-3^ |
|  |  |  |  |

| **Table S2** The BET surface area and pore size distribution analysis. |
| --- |

| **Surface Area** | **Pore Volume** | **Average Pore Diameter** |
| --- | --- | --- |
| 120.53 m^2^g^-1^ | 0.197 cm^3^g^-1^ | 6.53 nm |

**Table S3** Summary of photovoltaic performances of the FTO/TiO_2_ ETL/perovskite/spiro-OMeTAD/gold devices fabricated by various SILAR cycle under AM 1.5 illumination (100 mW cm^-2^).

| **ETL** | ***J_SC_* (mA cm^-2^)** | ***V_OC_* (V)** | ***ff* (%)** | **PCE (%)** |
| --- | --- | --- | --- | --- |
| TiO_2_- 10 cycle | 19.5 | 0.80 | 59.6 | 8.5 |
| **TiO_2_- 15 cycle** | **18.0** | **0.81** | **66.3** | **9.7** |
| TiO_2_- 20 cycle | 19.1 | 0.68 | 53.3 | 7.0 |

**Table S4** Comparative study of TiO_2_ with different synthesis method for perovskite solar cell application.

| **ETL** | **Phase** | **Morphology** | **Synthesis Method** | **PCE** | **References** |
| --- | --- | --- | --- | --- | --- |
| TiO_2_ | Rutile | Rod | Hydrothermal | 8.1 | 30 |
|  | Anatase | Spherical |  | 7.2 |  |
|  | Rutile | Nanorod | Hydrothermal | 9.4 | 31 |
|  | Rutile | Nanorod | Hydrothermal | 7.9 | 32 |
|  | NA | Irregular | Electrodeposition | 13.6 | 33 |
|  | Rutile | Nanograin | Chemical Bath Deposition | 11.1 | 34 |
|  | NA | Nanocrystal | Sol-gel | 11.4 | 35 |
|  | **Anatase** | **Spherical** | **SILAR** | **9.7** | **This work** |

**Table S5**: The comparison of reported sensor performances of different materials used for ammonia gas detection.

| **Sensor Material** | **Synthesis Method** | **C (ppm)** | **S (%)** | **T (°C)** | **Reference** |
| --- | --- | --- | --- | --- | --- |
| Functionalised SWCNT | Magnetron sputtering | 50 | 20.2 | 27 | 33 |
| MWCNT–PEDOT:PSS | Solution casting | 50 | 16 | 27 | 34 |
| CNT | Chemical vapour deposition | 100 | 1.5 | 27 | 35 |
| Graphene/polyaniline | Polymerisation | 100 | 10 | 27 | 36 |
| BAX-CS1% | Polymer-derived | 500 | 21 | 27 | 37 |
| **Anatase-TiO_2_** | **SILAR** | **100** | **80** | **27** | **This work** |

**Table S6:** Comparison of gas sensor responses based on TiO_2_ for different gas detection. (**C**-Concentration (ppm), **S**-Response (%), **T**-Temperature (°C)).

| **Sensor Material** | **Synthesis Method** | **Target gas** | **C (ppm)** | **S (%)** | **T (°C)** | **Reference** |
| --- | --- | --- | --- | --- | --- | --- |
| TiO_2_ Nanotubes | Anodization | H-CHO | 50 | 40 | 27 | 38 |
| TiO_2_ Nanotubes | Anodization | O_2_ | 200 | 100 | 50 | 39 |
| p-PANI/n-TiO_2_ | CBD/ED | LPG | 0.1 (Vol. %) | 60 | 27 | 40 |
| TiO_2_ | Anodization | H_2_ | 1000 | 170 | 150 | 41 |
| TiO_2_ Nanotubes | Anodization | CHCl_3_ | 1000 | 98.5 | 27 | 42 |
| **Anatase-TiO_2_** | **SILAR** | **NH_3_** | **100** | **80** | **27** | **This work** |

**Table S7** Photovoltaic parameters of FTO/TiO_2_ ETL/perovskite/spiro-OMeTAD/Au device as a function of scan rate and scan direction.

| **Scan rate (mV s^-1^)** | **Scan direction** | ***Jsc* (mA cm^-2^)** | ***Voc* (V)** | ***ff* (%)** | ***η* (%)** |
| --- | --- | --- | --- | --- | --- |
| 520 | Reverse | 18.7 | 0.83 | 58.2 | 8.5 |
|  | Forward | 18.3 | 0.81 | 48.3 | 6.8 |
| 52 | Reverse | 18.1 | 0.82 | 61.2 | 9.0 |
|  | Forward | 17.8 | 0.81 | 58.5 | 7.5 |
| 8.7 | Reverse | 18.2 | 0.81 | 66.3 | 9.7 |
|  | Forward | 18.1 | 0.81 | 60.1 | 9.1 |
